# The Bhopal disaster and its aftermath: a review

**DOI:** 10.1186/1476-069X-4-6

**Published:** 2005-05-10

**Authors:** Edward Broughton

**Affiliations:** 1 Columbia University, Mailman School of Public Health, 600 W 168th St. New York, NY 10032 USA

## Abstract

On December 3 1984, more than 40 tons of methyl isocyanate gas leaked from a pesticide plant in Bhopal, India, immediately killing at least 3,800 people and causing significant morbidity and premature death for many thousands more. The company involved in what became the worst industrial accident in history immediately tried to dissociate itself from legal responsibility. Eventually it reached a settlement with the Indian Government through mediation of that country's Supreme Court and accepted moral responsibility. It paid $470 million in compensation, a relatively small amount of based on significant underestimations of the long-term health consequences of exposure and the number of people exposed. The disaster indicated a need for enforceable international standards for environmental safety, preventative strategies to avoid similar accidents and industrial disaster preparedness.

Since the disaster, India has experienced rapid industrialization. While some positive changes in government policy and behavior of a few industries have taken place, major threats to the environment from rapid and poorly regulated industrial growth remain. Widespread environmental degradation with significant adverse human health consequences continues to occur throughout India.

## 

December 2004 marked the twentieth anniversary of the massive toxic gas leak from Union Carbide Corporation's chemical plant in Bhopal in the state of Madhya Pradesh, India that killed more than 3,800 people. This review examines the health effects of exposure to the disaster, the legal response, the lessons learned and whether or not these are put into practice in India in terms of industrial development, environmental management and public health.

## History

In the 1970s, the Indian government initiated policies to encourage foreign companies to invest in local industry. Union Carbide Corporation (UCC) was asked to build a plant for the manufacture of Sevin, a pesticide commonly used throughout Asia. As part of the deal, India's government insisted that a significant percentage of the investment come from local shareholders. The government itself had a 22% stake in the company's subsidiary, Union Carbide India Limited (UCIL) [1]. The company built the plant in Bhopal because of its central location and access to transport infrastructure. The specific site within the city was zoned for light industrial and commercial use, not for hazardous industry. The plant was initially approved only for formulation of pesticides from component chemicals, such as MIC imported from the parent company, in relatively small quantities. However, pressure from competition in the chemical industry led UCIL to implement "backward integration" – the manufacture of raw materials and intermediate products for formulation of the final product within one facility. This was inherently a more sophisticated and hazardous process [2].

In 1984, the plant was manufacturing Sevin at one quarter of its production capacity due to decreased demand for pesticides. Widespread crop failures and famine on the subcontinent in the 1980s led to increased indebtedness and decreased capital for farmers to invest in pesticides. Local managers were directed to close the plant and prepare it for sale in July 1984 due to decreased profitability [3]. When no ready buyer was found, UCIL made plans to dismantle key production units of the facility for shipment to another developing country. In the meantime, the facility continued to operate with safety equipment and procedures far below the standards found in its sister plant in Institute, West Virginia. The local government was aware of safety problems but was reticent to place heavy industrial safety and pollution control burdens on the struggling industry because it feared the economic effects of the loss of such a large employer [3].

At 11.00 PM on December 2 1984, while most of the one million residents of Bhopal slept, an operator at the plant noticed a small leak of methyl isocyanate (MIC) gas and increasing pressure inside a storage tank. The vent-gas scrubber, a safety device designer to neutralize toxic discharge from the MIC system, had been turned off three weeks prior [3]. Apparently a faulty valve had allowed one ton of water for cleaning internal pipes to mix with forty tons of MIC [1]. A 30 ton refrigeration unit that normally served as a safety component to cool the MIC storage tank had been drained of its coolant for use in another part of the plant [3]. Pressure and heat from the vigorous exothermic reaction in the tank continued to build. The gas flare safety system was out of action and had been for three months. At around 1.00 AM, December 3, loud rumbling reverberated around the plant as a safety valve gave way sending a plume of MIC gas into the early morning air [4]. Within hours, the streets of Bhopal were littered with human corpses and the carcasses of buffaloes, cows, dogs and birds. An estimated 3,800 people died immediately, mostly in the poor slum colony adjacent to the UCC plant [1,5]. Local hospitals were soon overwhelmed with the injured, a crisis further compounded by a lack of knowledge of exactly what gas was involved and what its effects were [1]. It became one of the worst chemical disasters in history and the name Bhopal became synonymous with industrial catastrophe [5].

Estimates of the number of people killed in the first few days by the plume from the UCC plant run as high as 10,000, with 15,000 to 20,000 premature deaths reportedly occurring in the subsequent two decades [6]. The Indian government reported that more than half a million people were exposed to the gas [7]. Several epidemiological studies conducted soon after the accident showed significant morbidity and increased mortality in the exposed population. Table 1. summarizes early and late effects on health. These data are likely to under-represent the true extent of adverse health effects because many exposed individuals left Bhopal immediately following the disaster never to return and were therefore lost to follow-up [8].

**Table 1:** Health effects of the Bhopal methyl isocyanate gas leak exposure [8, 30-32].

Early effects (0–6 months)	
Ocular	Chemosis, redness, watering, ulcers, photophobia
Respiratory	Distress, pulmonary edema, pneumonitis, pneumothorax.
Gastrointestinal	Persistent diarrhea, anorexia, persistent abdominal pain.
Genetic	Increased chromosomal abnormalities.
Psychological	Neuroses, anxiety states, adjustment reactions
Neurobehavioral	Impaired audio and visual memory, impaired vigilance attention and response time, Impaired reasoning and spatial ability, impaired psychomotor coordination.

Late effects (6 months onwards)	

Ocular	Persistent watering, corneal opacities, chronic conjunctivitis
Respiratory	Obstructive and restrictive airway disease, decreased lung function.
Reproductive	Increased pregnancy loss, increased infant mortality, decreased placental/fetal weight
Genetic	Increased chromosomal abnormalities
Neurobehavioral	Impaired associate learning, motor speed, precision

## Aftermath

Immediately after the disaster, UCC began attempts to dissociate itself from responsibility for the gas leak. Its principal tactic was to shift culpability to UCIL, stating the plant was wholly built and operated by the Indian subsidiary. It also fabricated scenarios involving sabotage by previously unknown Sikh extremist groups and disgruntled employees but this theory was impugned by numerous independent sources [1].

The toxic plume had barely cleared when, on December 7, the first multi-billion dollar lawsuit was filed by an American attorney in a U.S. court. This was the beginning of years of legal machinations in which the ethical implications of the tragedy and its affect on Bhopal's people were largely ignored. In March 1985, the Indian government enacted the Bhopal Gas Leak Disaster Act as a way of ensuring that claims arising from the accident would be dealt with speedily and equitably. The Act made the government the sole representative of the victims in legal proceedings both within and outside India. Eventually all cases were taken out of the U.S. legal system under the ruling of the presiding American judge and placed entirely under Indian jurisdiction much to the detriment of the injured parties.

In a settlement mediated by the Indian Supreme Court, UCC accepted moral responsibility and agreed to pay $470 million to the Indian government to be distributed to claimants as a full and final settlement. The figure was partly based on the disputed claim that only 3000 people died and 102,000 suffered permanent disabilities [9]. Upon announcing this settlement, shares of UCC rose $2 per share or 7% in value [1]. Had compensation in Bhopal been paid at the same rate that asbestosis victims where being awarded in US courts by defendant including UCC – which mined asbestos from 1963 to 1985 – the liability would have been greater than the $10 billion the company was worth and insured for in 1984 [10]. By the end of October 2003, according to the Bhopal Gas Tragedy Relief and Rehabilitation Department, compensation had been awarded to 554,895 people for injuries received and 15,310 survivors of those killed. The average amount to families of the dead was $2,200 [9].

At every turn, UCC has attempted to manipulate, obfuscate and withhold scientific data to the detriment of victims. Even to this date, the company has not stated exactly what was in the toxic cloud that enveloped the city on that December night [8]. When MIC is exposed to 200° heat, it forms degraded MIC that contains the more deadly hydrogen cyanide (HCN). There was clear evidence that the storage tank temperature did reach this level in the disaster. The cherry-red color of blood and viscera of some victims were characteristic of acute cyanide poisoning [11]. Moreover, many responded well to administration of sodium thiosulfate, an effective therapy for cyanide poisoning but not MIC exposure [11]. UCC initially recommended use of sodium thiosulfate but withdrew the statement later prompting suggestions that it attempted to cover up evidence of HCN in the gas leak. The presence of HCN was vigorously denied by UCC and was a point of conjecture among researchers [8,11-13].

As further insult, UCC discontinued operation at its Bhopal plant following the disaster but failed to clean up the industrial site completely. The plant continues to leak several toxic chemicals and heavy metals that have found their way into local aquifers. Dangerously contaminated water has now been added to the legacy left by the company for the people of Bhopal [1,14].

## Lessons learned

The events in Bhopal revealed that expanding industrialization in developing countries without concurrent evolution in safety regulations could have catastrophic consequences [4]. The disaster demonstrated that seemingly local problems of industrial hazards and toxic contamination are often tied to global market dynamics. UCC's Sevin production plant was built in Madhya Pradesh not to avoid environmental regulations in the U.S. but to exploit the large and growing Indian pesticide market. However the manner in which the project was executed suggests the existence of a double standard for multinational corporations operating in developing countries [1]. Enforceable uniform international operating regulations for hazardous industries would have provided a mechanism for significantly improved in safety in Bhopal. Even without enforcement, international standards could provide norms for measuring performance of individual companies engaged in hazardous activities such as the manufacture of pesticides and other toxic chemicals in India [15]. National governments and international agencies should focus on widely applicable techniques for corporate responsibility and accident prevention as much in the developing world context as in advanced industrial nations [16]. Specifically, prevention should include risk reduction in plant location and design and safety legislation [17].

Local governments clearly cannot allow industrial facilities to be situated within urban areas, regardless of the evolution of land use over time. Industry and government need to bring proper financial support to local communities so they can provide medical and other necessary services to reduce morbidity, mortality and material loss in the case of industrial accidents.

Public health infrastructure was very weak in Bhopal in 1984. Tap water was available for only a few hours a day and was of very poor quality. With no functioning sewage system, untreated human waste was dumped into two nearby lakes, one a source of drinking water. The city had four major hospitals but there was a shortage of physicians and hospital beds. There was also no mass casualty emergency response system in place in the city [3]. Existing public health infrastructure needs to be taken into account when hazardous industries choose sites for manufacturing plants. Future management of industrial development requires that appropriate resources be devoted to advance planning before any disaster occurs [18]. Communities that do not possess infrastructure and technical expertise to respond adequately to such industrial accidents should not be chosen as sites for hazardous industry.

## Since 1984

Following the events of December 3 1984 environmental awareness and activism in India increased significantly. The Environment Protection Act was passed in 1986, creating the Ministry of Environment and Forests (MoEF) and strengthening India's commitment to the environment. Under the new act, the MoEF was given overall responsibility for administering and enforcing environmental laws and policies. It established the importance of integrating environmental strategies into all industrial development plans for the country. However, despite greater government commitment to protect public health, forests, and wildlife, policies geared to developing the country's economy have taken precedence in the last 20 years [19].

India has undergone tremendous economic growth in the two decades since the Bhopal disaster. Gross domestic product (GDP) per capita has increased from $1,000 in 1984 to $2,900 in 2004 and it continues to grow at a rate of over 8% per year [20]. Rapid industrial development has contributed greatly to economic growth but there has been significant cost in environmental degradation and increased public health risks. Since abatement efforts consume a large portion of India's GDP, MoEF faces an uphill battle as it tries to fulfill its mandate of reducing industrial pollution [19]. Heavy reliance on coal-fired power plants and poor enforcement of vehicle emission laws have result from economic concerns taking precedence over environmental protection [19].

With the industrial growth since 1984, there has been an increase in small scale industries (SSIs) that are clustered about major urban areas in India. There are generally less stringent rules for the treatment of waste produced by SSIs due to less waste generation within each individual industry. This has allowed SSIs to dispose of untreated wastewater into drainage systems that flow directly into rivers. New Delhi's Yamuna River is illustrative. Dangerously high levels of heavy metals such as lead, cobalt, cadmium, chrome, nickel and zinc have been detected in this river which is a major supply of potable water to India's capital thus posing a potential health risk to the people living there and areas downstream [21].

Land pollution due to uncontrolled disposal of industrial solid and hazardous waste is also a problem throughout India. With rapid industrialization, the generation of industrial solid and hazardous waste has increased appreciably and the environmental impact is significant [22].

India relaxed its controls on foreign investment in order to accede to WTO rules and thereby attract an increasing flow of capital. In the process, a number of environmental regulations are being rolled back as growing foreign investments continue to roll in. The Indian experience is comparable to that of a number of developing countries that are experiencing the environmental impacts of structural adjustment. Exploitation and export of natural resources has accelerated on the subcontinent. Prohibitions against locating industrial facilities in ecologically sensitive zones have been eliminated while conservation zones are being stripped of their status so that pesticide, cement and bauxite mines can be built [23]. Heavy reliance on coal-fired power plants and poor enforcement of vehicle emission laws are other consequences of economic concerns taking precedence over environmental protection [19].

In March 2001, residents of Kodaikanal in southern India caught the Anglo-Dutch company, Unilever, red-handed when they discovered a dumpsite with toxic mercury laced waste from a thermometer factory run by the company's Indian subsidiary, Hindustan Lever. The 7.4 ton stockpile of mercury-laden glass was found in torn stacks spilling onto the ground in a scrap metal yard located near a school. In the fall of 2001, steel from the ruins of the World Trade Center was exported to India apparently without first being tested for contamination from asbestos and heavy metals present in the twin tower debris. Other examples of poor environmental stewardship and economic considerations taking precedence over public health concerns abound [24].

The Bhopal disaster could have changed the nature of the chemical industry and caused a reexamination of the necessity to produce such potentially harmful products in the first place. However the lessons of acute and chronic effects of exposure to pesticides and their precursors in Bhopal has not changed agricultural practice patterns. An estimated 3 million people per year suffer the consequences of pesticide poisoning with most exposure occurring in the agricultural developing world. It is reported to be the cause of at least 22,000 deaths in India each year. In the state of Kerala, significant mortality and morbidity have been reported following exposure to Endosulfan, a toxic pesticide whose use continued for 15 years after the events of Bhopal [25].

Aggressive marketing of asbestos continues in developing countries as a result of restrictions being placed on its use in developed nations due to the well-established link between asbestos products and respiratory diseases. India has become a major consumer, using around 100,000 tons of asbestos per year, 80% of which is imported with Canada being the largest overseas supplier. Mining, production and use of asbestos in India is very loosely regulated despite the health hazards. Reports have shown morbidity and mortality from asbestos related disease will continue in India without enforcement of a ban or significantly tighter controls [26,27].

UCC has shrunk to one sixth of its size since the Bhopal disaster in an effort to restructure and divest itself. By doing so, the company avoided a hostile takeover, placed a significant portion of UCC's assets out of legal reach of the victims and gave its shareholder and top executives bountiful profits [1]. The company still operates under the ownership of Dow Chemicals and still states on its website that the Bhopal disaster was "cause by deliberate sabotage". [28].

Some positive changes were seen following the Bhopal disaster. The British chemical company, ICI, whose Indian subsidiary manufactured pesticides, increased attention to health, safety and environmental issues following the events of December 1984. The subsidiary now spends 30–40% of their capital expenditures on environmental-related projects. However, they still do not adhere to standards as strict as their parent company in the UK. [24].

The US chemical giant DuPont learned its lesson of Bhopal in a different way. The company attempted for a decade to export a nylon plant from Richmond, VA to Goa, India. In its early negotiations with the Indian government, DuPont had sought and won a remarkable clause in its investment agreement that absolved it from all liabilities in case of an accident. But the people of Goa were not willing to acquiesce while an important ecological site was cleared for a heavy polluting industry. After nearly a decade of protesting by Goa's residents, DuPont was forced to scuttle plans there. Chennai was the next proposed site for the plastics plant. The state government there made significantly greater demand on DuPont for concessions on public health and environmental protection. Eventually, these plans were also aborted due to what the company called "financial concerns". [29].

## Conclusion

The tragedy of Bhopal continues to be a warning sign at once ignored and heeded. Bhopal and its aftermath were a warning that the path to industrialization, for developing countries in general and India in particular, is fraught with human, environmental and economic perils. Some moves by the Indian government, including the formation of the MoEF, have served to offer some protection of the public's health from the harmful practices of local and multinational heavy industry and grassroots organizations that have also played a part in opposing rampant development. The Indian economy is growing at a tremendous rate but at significant cost in environmental health and public safety as large and small companies throughout the subcontinent continue to pollute. Far more remains to be done for public health in the context of industrialization to show that the lessons of the countless thousands dead in Bhopal have truly been heeded.

## Competing interests

The author(s) declare that they have no competing interests.
